# 1720. *In Vitro* Activity of Aztreonam-Avibactam and Comparator Agents Against Enterobacterales from Patients with Urinary Tract Infections Collected During the ATLAS Global Surveillance Program, 2017-2020

**DOI:** 10.1093/ofid/ofac492.1350

**Published:** 2022-12-15

**Authors:** Mark Estabrook, Francis Arhin, Daniel F Sahm

**Affiliations:** IHMA, Schaumburg, Illinois; Pfizer, Inc., Kirkland, Manitoba, Canada; IHMA, Schaumburg, Illinois

## Abstract

**Background:**

β-lactamase-producing Enterobacterales (Eba) frequently carry resistance mechanisms for multiple drug classes, limiting treatment options. Avibactam (AVI) inhibits class A, class C, and some class D serine β-lactamases, while aztreonam (ATM) is refractory to hydrolysis by class B metallo-β-lactamases (MBLs). ATM-AVI is being developed for use against drug-resistant isolates of Eba, especially those co-producing MBLs and serine β-lactamases. This study evaluated the *in vitro* activity of ATM-AVI and comparators against Eba collected in 2017-2020 from patients with urinary tract infections (UTI) as part of the ATLAS global surveillance program.

**Methods:**

Non-duplicate clinical isolates were collected from 239 sites in 55 countries in Europe, Latin America, Asia/Pacific (excluding mainland China and India), and Middle East/Africa. Susceptibility testing was performed by CLSI broth microdilution and interpreted using CLSI 2022 and FDA (tigecycline) breakpoints. PCR and sequencing were used to determine the β-lactamase genes present in all isolates with meropenem MIC >1 µg/mL, and *Escherichia coli*, *Klebsiella* spp. and *Proteus mirabilis* with ATM or ceftazidime MIC >1 µg/mL.

**Results:**

Based on MIC_90_ values, ATM-AVI was at least as active as every comparator agent tested against all 15085 Eba isolates collected from UTI (0.12 µg/ml; table), with only five isolates testing with MIC values >8 µg/ml (not shown). Against resistant subsets of isolates, MIC_90_ values for ATM-AVI were 0.25-0.5 µg/ml, 4- to 8-fold lower than any comparator tested. Against MBL-positive isolates, ATM-AVI MIC values ranged from ≤0.015-2 µg/ml with the exception of one *Escherichia coli* isolate testing with an MIC value of 16 µg/ml that carried NDM-5, CMY-145, CTX-M-55 and TEM (not shown).

**Figure:**
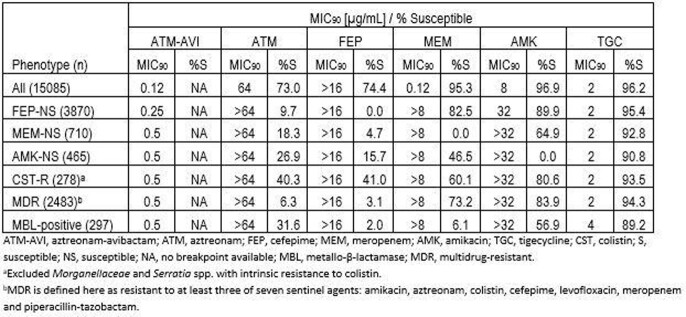

**Conclusion:**

Based on MIC_90_ values, ATM-AVI was the most potent agent tested against drug-resistant and MBL-positive subsets of Eba collected from UTI. Based on the potent *in vitro* activity of ATM-AVI, continued development of this combination for treatment of UTI caused by drug-resistant Eba is warranted.

**Disclosures:**

**All Authors**: No reported disclosures.

